# Effect of Waiting Period on Initial Adverse Vaginal Microbiome Composition in IVF-ICSI Patients

**DOI:** 10.3390/jcm13175024

**Published:** 2024-08-25

**Authors:** Alexandra Petra Bielfeld, Dunja Maria Baston-Buest, Philippos Edimiris, Jan-Steffen Kruessel

**Affiliations:** Department of Obstetrics/Gynecology and Reproductive Endocrinology and Infertility (UniKiD), University of Duesseldorf Medical Center, Moorenstrasse 5, 40225 Duesseldorf, Germany; alexandra.bielfeld@med.uni-duesseldorf.de (A.P.B.); dunja.baston-buest@med.uni-duesseldorf.de (D.M.B.-B.); jan-steffen.kruessel@med.uni-duesseldorf.de (J.-S.K.)

**Keywords:** vaginal microbiome, assisted reproduction, IVF-ICSI, *Lactobacillus crispatus*, add-on

## Abstract

**Background/Objectives**: In this observational prospective cohort study, conducted at the Fertility Centre of the University Hospital, Duesseldorf Germany, the spontaneous reversal capacity and the effect of waiting time on an adverse vaginal microbiome profile in subfertile patients were investigated. **Methods**: Vaginal swabs of 76 patients were obtained before starting a fertility treatment using a commercially available test to perform a microbiome analysis. Patients with a favorable microbiome profile (“medium” or “high profile”) according to the manufacturer’s algorithm proceeded with the fertility treatment. Patients with an unfavorable microbiome profile (“low profile”) postponed their fertility treatment and were sampled up to four times in each successive cycle or until a shift to a more favorable profile was detected. **Results**: Initially, 54/76 subjects had a high or medium profile and 23/76 had a low profile. Within 3 months, 75% of patients with an initial low profile shifted to a more favorable profile (7/23 dropouts). The presence of *Lactobacillus crispatus* in the initial sample was associated with a higher likelihood of a spontaneous shift to a more favorable profile. **Conclusions**: The vaginal microbiome is subject to strong fluctuations. Even an unfavorable microbiome profile can develop into a favorable microbiome profile within a few months without treatment.

## 1. Introduction

Over the past decade, the microbiome has increasingly attracted the attention of research, especially since the completion of the Human Microbiome Project [1]. It was demonstrated that it is heavily involved in the role of maintaining health and establishing diseases [2,3]. Although the microbiome in the female reproductive tract has been less explored so far, recently a similar trend could be observed [4,5]. It was shown already that bacteria inhabiting the vagina and the reproductive tract mostly belong to the genus *Lactobacillus* [6]. Other represented genera are Prevotella, Bifidobacterium, Gardnerella, Atopobium, Megasphaera, Sneathia, and Anaerococcus [1,7]. Furthermore, it is known that the presence of *Lactobacilli* has a positive influence on the outcome of assisted reproductive technology (ART) such as in vitro fertilization (IVF) and intracytoplasmic sperm injection (ICSI) [8,9,10].

Recently, a commercially available test was established which predicts the receptivity of the individual’s endometrium to accept an embryo for implantation based on the vaginal microbiome assessed before the onset of IVF treatment [11]. It has been validated in a multicenter study in the Netherlands, with external validation in a German IVF clinic, showing a high predictive accuracy and specificity [11,12]. The available test enables a disposition of the chances for subsequent IVF success in three stratification groups: low, with a 5.9% success rate; medium, with 23.8%; and high, with a 52.6% success rate for achieving a clinical pregnancy, as confirmed by ultrasound examination.

The data obtained so far suggest that the test has the potential to be used as a personalized timing tool in routine IVF to increase pregnancy chances. However, the temporal dynamics of the vaginal microbiota have been scarcely investigated so far and, if so, have rather been tested in women of reproductive age in general instead of IVF patients. A better understanding of the dynamics of an adverse profile is of enormous importance to counsel IVF patients adequately. Therefore, we aimed to find out whether women with a low profile according to the test shift to another stratification over time without a particular treatment. To answer this question, vaginal swabs for the analysis of the microbiome from individuals prior to their IVF, IVF-ICSI, or frozen–thawed embryo transfer (FET) cycle were collected prospectively. Low-profile individuals were asked to postpone their treatment until the next cycle and would only proceed with the treatment if a profile shift to medium or high occurred.

## 2. Materials and Methods

Ethical approval was obtained in accordance with the Declaration of Helsinki (6259R MPG§23b) by the Heinrich Heine University Duesseldorf Ethical Board. Patients eligible for IVF/IVF-ICSI/FET cycle treatment were recruited in the UniKiD- Department of OB/GYN and REI, University Hospital of Duesseldorf, Germany. Informed consent was obtained from all participants. The subject recruitment was carried out from February 2019 to September 2020. 

### 2.1. Assessing the Individual Vaginal Microbiome

Vaginal swabs of all patients were taken after obtaining informed consent, and patients with a low profile were subsequently sampled each following month until a shift occurred. Sampling was carried out with the ReceptIVFITY^®^ test kit (ARTPred B.V., 1438 AX Oude Meer, The Netherlands), following the manufacturer’s instructions as described earlier [11,12], using the supplied vaginal swab, storing the swab in the supplied reduced transport fluid (RTF) buffer and shipped frozen to the laboratory where the microbiome was analyzed using the intergenic spacer (IS)-pro technique [11]. IS-pro is a eubacterial technique based on the detection and categorization of the length of the 16S–23S ribosomal ribonucleic acid (rRNA) gene interspace region according to the manufacturers’ instruction [11]. The following bacteria are specified in the report: *Lactobacillus* (L.) *crispatus*, *L. iners*, *L. jensenii*, L. spp., *Gardnerella vaginalis*, Proteobacteria. The further bacteria found are summarized as “others”. The individuals’ vaginal microbiome was stratified into three different groups: low (relative *Lactobacillus* load < 20% and/or *L. jensenii* > 35% and/or presence of *Gardnerella vaginalis* Interspace Type 1 (IST1) and/or Proteobacteria > 28% of total bacterial load), medium (not low and *L. crispatus* ≥ 60%) and high (not low and *L. crispatus* < 60%) [12]. 

### 2.2. Study Design

The analysis took place before the start of the IVF/IVF-ICSI/FET cycle treatment. Patients with a high or medium stratification proceeded directly to the start of the IVF/IVF-ICSI/FET procedure. However, patients with a low stratification were put on hold and were tested again on a monthly basis (for a maximum of 4 months) or until their profile changed to medium or high. As soon as patients that initially showed a low profile shifted to medium or high stratifications, the IVF/IVF-ICSI/FET procedure was started.

The study design did not incorporate the use of pro- or antibiotics. Patients were advised accordingly and gave informed consent regarding this matter. 

### 2.3. Study Population

Inclusion criteria were the following: women aged between 18 and 44 years (minimum age of the study population was 24 years and maximum age was 42 years); indication for an IVF or IVF/ICSI or FET procedure present; no preimplantation genetic testing; women eligible for their first, second, or third IVF or IVF/ICSI attempt; willingness to provide multiple vaginal swabs; willingness to provide informed consent; willingness to postpone the ART attempt for a maximum of 5 consecutive months (according to four cycles) when a low scoring vaginal microbiome profile is observed.

### 2.4. Statistical Analysis

Statistical analysis was performed by using a *t*-test for not paired groups (Microsoft Excel 2016). A *p*-value of *p* < 0.05 was considered statistically significant. To determine the percentage of shifters in time, a correction for dropouts was performed.

## 3. Results

**Figure 1:**
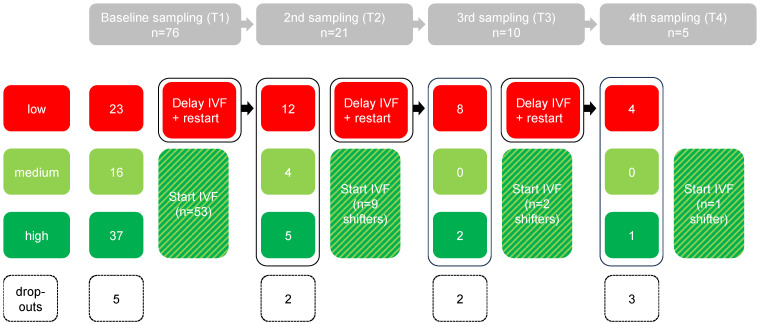
Study flow chart with data follow-up of all patients displaying the 3 different analysis scores (low (red), medium (light green), and high (dark green)) and the course of each patient including dropouts.

In total, 81 women were included with the intention to provide a vaginal swab for bacterial profile analysis. Three women did not provide a vaginal swab, and two women were excluded because the sample was lost during transport (Figure 1). Of the remaining dataset (n = 76), the distribution of initial test results was as follows: 53 with a high or medium profile (70%) and 23 with a low profile (30%) (Figure 1).

Baseline aspects of the different groups are depicted in Table 1, showing the absence of statistical deviations regarding body mass index (BMI) and age between patients with an initial high/medium score vs. shifters from an initial low score (Table 1).

### 3.1. Results of the Serial Examination

The serial examination was performed within the group of subjects with an initial low profile (n = 23) (Figure 1). Of these patients, after the first, second, and third month, two, two, and three subjects, respectively, resulting in a total of seven subjects (30%), dropped out.

**Figure 2:**
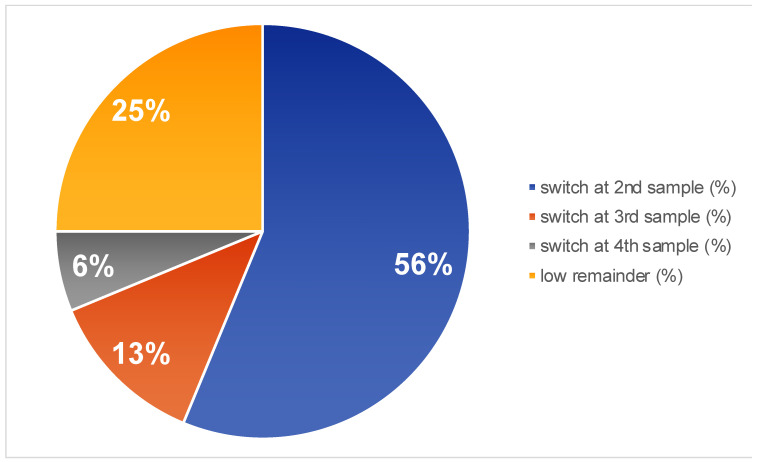
Percentage of shifters from an initial low score to a medium/high score over time.

Looking at the profiles that were initially identified as low (without the drop outs, n = 16), it was observed that after the second/third and fourth assessment, 9/16 (56%), 2/16 (13%), and 1/16 (6%) changed to a more favorable profile, respectively (Figure 2). Only 4/16 (25%) of the initial low profiles remained low (Figure 2).

### 3.2. Microbiome Compostition of the Low Profiles

**Figure 3:**
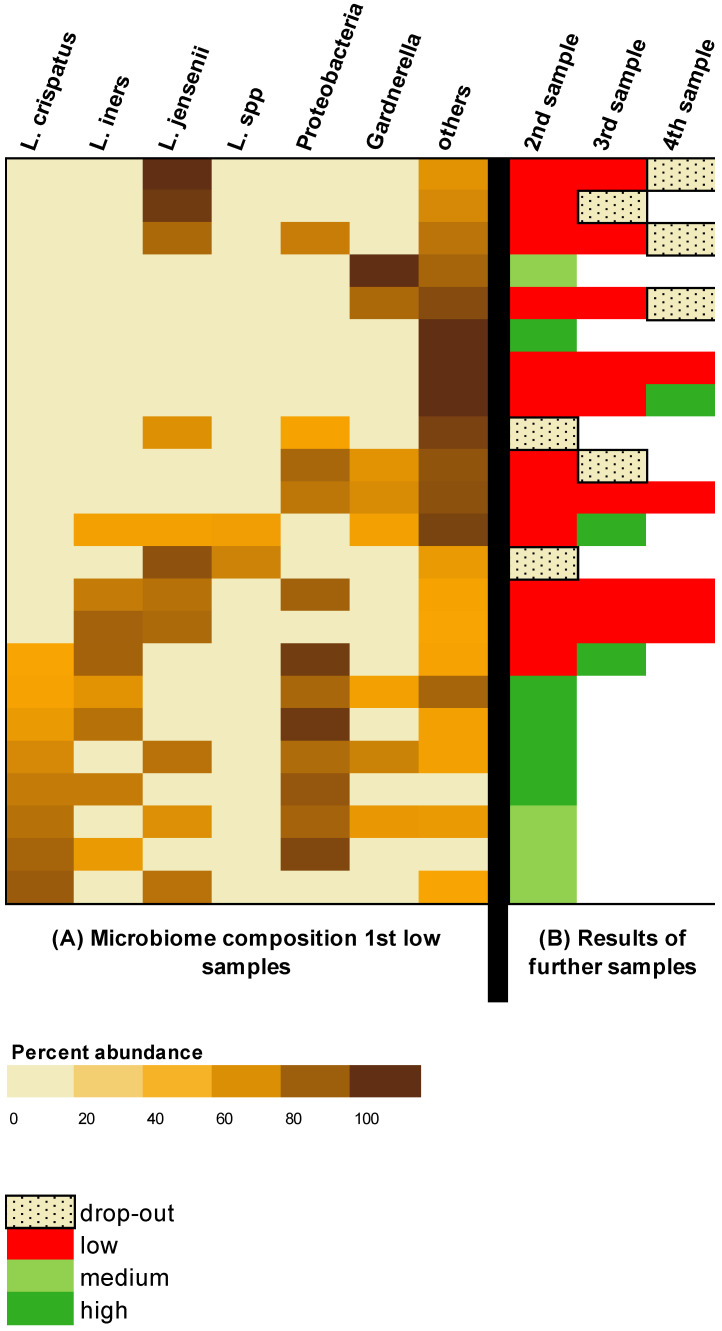
(**A**) Heatmap displaying the microbiome composition of each of the subjects (n = 23) with an initial low score according to the test. (**B**) Microbiome test results of the 23 subjects studied serially.

The bacterial composition of the subjects with an initial low result and the result of the serial examination are shown in Figure 3. In a total of 7/23 (30%) subjects, no *Lactobacilli* could be found at all. *L. crispatus* and *L. iners* were present in 8/23 (35%) subjects, respectively. *L. jensenii* was detected in 11/23 (48%) subjects. Gardnerella was present in 8/23 (35%) patients.

All eight subjects in whom the initial sample contained *L. crispatus* switched to a more favorable microbiome profile in the follow-up. In 7/8 (88%) of these subjects, the change was evident in the second sample; in 1/8 (13%) subjects, the change was seen in the third sample. There was no study dropout in this group of patients with *L. crispatus* in the first sample.

This should be distinguished from the group consisting of 15 subjects whose initial sample did not show *L. crispatus*; out of this group, 4/15 (27%) subjects switched to a more favorable profile. The average number of samples per subject was 2.3. However, 7/15 (47%) of theses subjects terminated the study before completion.

In the serial examination of subjects with initial detection of *L. jensenii* (n = 11), a change was detected in only four subjects (besides two dropouts). All of these changes occurred in subjects whose initial samples also contained *L. crispatus* or *L. iners*. A total of five subjects whose initial sample did not contain any other *Lactobacilli* except *L. jensenii* did not show any changes over the investigated period. 

In contrast, three of the seven subjects whose sample initially contained no *Lactobacilli* showed a change to a more favorable microbiome in the serial examination. 

In 5/8 (63%) subjects with Gardnerella in the initial sample, Gardnerella was no longer detected during the follow-up and a switch to a more favorable microbiome was achieved. In these five subjects in whom Garnerella vaginalis disappeared in the serial examination, *Lactobacilli* were present in the initial sample.

**Figure 4:**
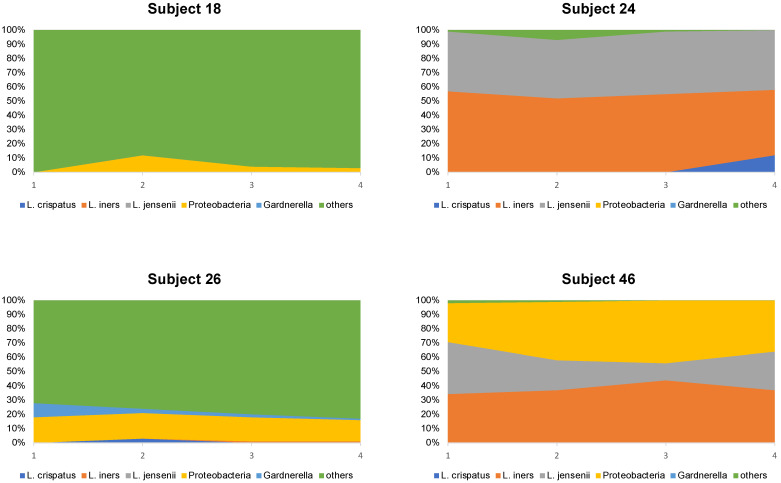
Serial microbiome composition of the 4 subjects, who consistently had a low score according to the microbiome test.

Looking at the microbiome composition of the four subjects who continued to have a poor profile after 4 months (subjects 18, 24, 26, and 46), it was observed that the microbiome composition hardly changed within the study period (Figure 4).

A detailed presentation of the reproductive outcomes of the study can be found in Supplementary Table S1.

## 4. Discussion

The results of this study provide valuable insights into the dynamics of the vaginal microbiome and its potential for change over time. So far, the temporal dynamics of the vaginal microbiome were only investigated in healthy, fertile woman but not in patients in an ART setting seeking therapy for their involuntary childlessness. However, since altered vaginal microbiota and bacterial vaginosis may be related to poor pregnancy outcomes, patients undergoing ART should be screened and eventually treated to enhance the chances of success [13]. Moreover, the vaginal microbiome also plays a crucial role in fetal programming, as it influences the initial colonization of the newborn’s microbiome, which is essential for the development of a healthy immune system. Therefore, ensuring and testing for a balanced vaginal microbiome can provide significant benefits for both the mother and her unborn child [14].

Screening of the vaginal microbiome in this study was performed shortly before a planned embryo transfer. The test results differed with low, medium, and high scores according to the manufacturer’s algorithm. In patients with a medium or high score, embryo transfer was performed as planned. In the case of a low score, serial analysis of the vaginal microbiome was performed every 4 weeks up to 4 times or until a medium or high profile was achieved.

### 4.1. Fluctuations of the Vaginal Microbiome

The study revealed that among the initial low-profile subjects, a significant proportion experienced a transition to a more favorable vaginal microbiome. After the second, third, and fourth assessments, 56%, 13%, and 6% of the women, respectively, exhibited improved profiles. The observation that the vaginal microbiome is not static and changes in bacterial composition occur without medical involvement solely by time is consistent with another study examining the temporal dynamics of the vaginal mirobiome [15]. In this study, in which the vaginal microbiome was longitudinally monitored in five clusters called community state types (CSTs), stability ranged from 56% (CST IV-A) to 84 to 89% (all other CST types) [15]. However, one cannot compare the data, especially since the population of the study by Gajer et al. consists of healthy subjects rather than subfertile patients. For example, none of the 32 patients examined had a microbiome dominated by *L. jensenii*, whereas in our population a total of 11 samples contained *L. jensenii*, among which *L. jensenii* was the dominant *Lactobacillus* in 7 samples. However, *L. jensenii*, if it is the dominant lactobacillus strain, may be associated with a lower reproductive outcome [12].

### 4.2. The Role of L. crispatus

Interestingly, the presence of *L. crispatus* in the initial sample was associated with a higher likelihood of preferable microbiome changes. All eight subjects in whom *L. crispatus* was detected experienced a switch to a more favorable microbiome composition during the follow-up period, with 88% already experiencing a switch after 1 month. The existence of *L. crispatus* possibly increases the self-healing capacity in the presence of dysbiosis. 

In contrast, among the subjects whose initial sample did not contain *L. crispatus*, only a minority experienced a switch to a more favorable microbiome result. This suggests that the absence of *L. crispatus* may be associated with a less resilient microbiome, making it more challenging to achieve positive shifts in bacterial composition.

Now, relating this information to the infertile patient, it could be possible that the absence of *L. crispatus* is a reason for repeated implantation failure. This assumption is based on the observation that the profiles with *L. crispatus* changed to a better profile in a short time, while the profiles without *L. crispatus* remained unfavorable in a high percentage of patients. This hypothesis is further supported by the fact that the detection of *L. crispatus* in the vaginal and/or endometrial microbiome may have a positive impact on reproductive outcome [16,17,18].

However, since the observation in this study was not longer than 4 months and patients did not suffer from recurrent implantation failure, this theory cannot be verified by our data.

Future research should further explore the factors influencing the presence or absence of *L. crispatus* and its implications for vaginal and reproductive health.

### 4.3. Gardnerella as a Transient Bacterium

Another important finding of this study was the disappearance of Gardnerella in the follow-up assessments of five subjects whose initial sample contained Gardnerella. This suggests that Gardnerella may not only be a pathogen but also a transient bacterium that can be eliminated or suppressed over time, leading to a more favorable microbiome composition, especially if the microbiome contains *Lactobacilli* [19].

### 4.4. Strengths and Limitations

A limitation might be that a considerable number of participants dropped out of the study before completion, which may introduce biases and limit the generalizability of the findings. Within this context, it is plausible that the high dropout rate can be attributed to the deferment of IVF therapy. Given that individuals desiring children often experience subjective time constraints, it is conceivable that some participants prematurely discontinued their involvement in the study. Another limitation might be that it cannot be guaranteed that patients with a low profile initiated lifestyle changes (e.g., diet, vaginal hygiene) that led to the high conversion rate.

The strengths of the study are that the samples were taken by medical personnel, that the study period was very long, and that the anaylsis was performed in a population of patients with an unfulfilled desire to have children.

### 4.5. Conclusions

In conclusion, this study highlights the dynamic nature of the vaginal microbiome and its potential for change over time. The findings suggest that individuals with initially low bacterial profiles have a significant likelihood of transitioning to a more favorable microbiome composition. The presence of *L. crispatus* in the initial sample was associated with a higher probability of positive microbiome changes, indicating its potential importance in maintaining vaginal health. Additionally, the disappearance of Gardnerella in follow-up assessments suggests that this bacterium may also be transient and its elimination could lead to improved female reproductive health. In the context of patients about to undergo IVF therapy, the vaginal microbiome test investigated could help to evaluate the optimal timing for starting IVF therapy. Future research should focus on the impact of the vaginal microbiome on the outcome of IVF therapy and explore therapeutic options to achieve a better microbiome, especially in patients who do not have a high likelihood of changing the microbiome.

## Figures and Tables

**Table 1:** Demographic characteristics of the study group were divided into 4 groups (initial high/med, shift in the 1st month, 2nd month, and 3rd month). No statistically significant difference (*p* < 0.05) could be observed. ^a^ *t* test.

	Initial High/Medium (a)	1st Month Shift (b)	2nd Month Shift (c)	3rd Month Shift	*p* Values		
n	54	9	2	1	a vs. b	a vs. c	b vs. c
body mass index, kg/m^2^	23.5 ± 3.8	23.3 ± 3.6	24.3 ± 3.5		0.92 ^a^	0.74 ^a^	0.71 ^a^
age in years	34.8 ± 0.73	34.5 ± 3.1	32 ± 2.7		0.94 ^a^	0.94 ^a^	0.26 ^a^

## Data Availability

The datasets generated and analyzed during the current study are available from the corresponding author on reasonable request. With the IS-pro technique, which we used in the study, we do not produce sequence data, so there are no DNA or RNA sequences to share. The analyzed IS-pro data are already featured in the paper.

## Supplementary Materials

The following supporting information can be downloaded at: https://www.mdpi.com/article/10.3390/jcm13175024/s1, Table S1: Reproductive outcomes of the study.

## Abbreviations

ART: assisted reproductive technology, IVF: in vitro fertilization, ICSI: intracytoplasmic sperm injection, RTF: reduced transport fluid, IS intergenic spacer, rRNA: ribosomal ribonucleic acid, NGS: next-generation sequencing, L.: Lactobacillus, IST1: Interspace Type 1, FET: frozen–thawed embryo transfer, BMI: body mass index, CST: community state type.
